# Formation of gigahertz pulse train by chirped terahertz pulses interference

**DOI:** 10.1038/s41598-020-66437-4

**Published:** 2020-06-11

**Authors:** Xinrui Liu, Maksim Melnik, Maria Zhukova, Egor Oparin, Joel J. P. C. Rodrigues, Anton Tcypkin, Sergei Kozlov

**Affiliations:** 1 International Laboratory of Femtosecond Optics and Femtotechnologies, ITMO University, St. Petersburg, 197101 Russia; 2 Federal University of Piauí, (UFPI), Teresina, Pi 64049-550 Brazil; 3 Instituto de Telecomunicações, 1049-001 Lisboa, Portugal

**Keywords:** Terahertz optics, Ultrafast photonics

## Abstract

The state-of-art broadband THz sources can contribute to the development of short-range 6G communications. This paper has demonstrated the feasibility of forming the controllable sequence of THz subpulses in the temporal domain and the corresponding quasidiscrete spectrum by the interference of two THz pulses with an exponential chirp. Moreover, due to small time delay between these pulses the temporal and spectral structures are similar to each other (so-called “linkage relation”). This will benefit information encoding in the THz range. The calculated metrics for the prototype communication channel based on the proposed method are competitive with existing short-range THz CW channels.

## Introduction

The maturity and commercialization of the fifth generation (5G) has already arrived^1^. 6G (6th generation wireless systems) – a technology that supposed 100 to 1000 times^2,3^ faster than 5G, is considered to come to our lives in the next ten years^4^. Fresh spectral bands as well as advanced physical layer solutions are required for future wireless communications and THz wireless technology is a potential candidate^5–9^. Usually optical frequency combs are used in IR optical communication systems providing high data rates for long distances^10^. In this case, a large number of channels can be created using spectrum-sliced supercontinuum (SSSC) by arranging the interference of the two pulses with ultra-broadband spectra^11,12^. In the THz range a typical method to generate THz spectral comb is THz generator pumped by a pulse train of fs laser pulses^13^. It has also been reported that the transmitted spectrum of a THz pulse through a multilayer filter could achieve sub-comb structure^14^. As a THz frequency comb, it can be used to measure the frequency of a continuous-wave THz with high precision^15^; is suitable for the source of THz wireless communication of wavelength division multiplexing^16^, and expected to be used in ultra-fast information transmission systems^17^. The use of pulsed THz sources is limited by strong free space losses due to the wide spectrum which does not overlap with atmospheric transparency window^18^. Therefore, THz links have been suggested to use pulsed sources for short distance indoor communications^19,20^, and to use continuous radiation sources for long distances^21,22^. The implementation of these techniques relies on ultra-fast modulation as well as encoding and decoding methods of THz wave, in which pulsed broadband THz technologies can make contributions^23^.

In the case of pulsed broadband radiation, the main aim is to create encoding methods based on wavelength division multiplexing (WDM). For instance, quasi-discrete THz supercontinuum, obtained via spectral interference of two THz pulses, can be used to achieve a data transfer rate of 34.1 Gb/s with 31 THz spectral lines^24^. In the NIR region, the profiling of the spectral structure and the corresponding pulse sequence with a THz repetition rate has been verified both experimentally and analytically^25–27^. The peculiarity of this approach lies in the fact that there is the pulse train corresponding to the profiling spectral structure (“linkage relation”) which helps to preserve transmitted data from corruption. In the case of pulsed THz radiation, which consists of a small number of oscillations^28^, the formation of these structures by aforementioned method occurs when the following conditions are met: the THz pulses should be carefully chirped and the time delay between them is shorter than their duration. In the THz range, chirping and pulse elongation can be accomplished by using hollow metal waveguides^29^.

In this paper, we show for the first time the possibility of forming a frequency comb and the corresponding temporal pulses sequence in THz frequency range. The “linkage relation” between the emerging temporal and spectral structures is shown. Thus, changes in spectrum will lead to similar changes in temporal domain. Using proposed method data rate of 225 Mbit/sec can be achieved. The communication channel based on this method is comparable with existing short-range THz CW channels^30–33^. However these results can be improved by implementing pump laser with higher repetition rate and adjusting the interferometer. This technique allows to create short-range communication network and devices which can operate at room temperature.

## Results

**Figure 1:**
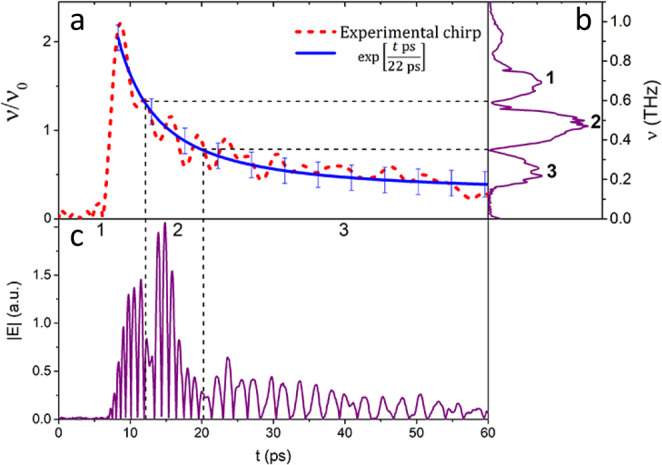
(**a**) Experimental chirp of THz pulse and its exponential approximation, (**b**) quasi-discrete spectrum (**c**) and temporal structure of the pulse train.

The experimental part of the work was carried out on a setup consisting of a THz spectrometer with a Michelson interferometer (see Methods). The interferometer was used to form two fs pulses with a variable delay between them of a longer duration. The obtained fs pulses generated THz pulses by photo-Dember effect in InAs crystal. THz pulses, in turn, propagated in a hollow metal waveguide chirping and interfering. The presence of the chirp enables to observe not only the formation of a quasi-discrete spectrum^24^, but also the formation of a train of pulses during the interference^26,27^. This is possible due to the correct selection of conditions, namely, a small delay between pulses and the presence of a chirp with a known dependence. The chirp of experimentally obtained THz pulses is shown in Fig. 1(a) (red curve). As can be seen, it is well approximated by an exponential function (blue curve). THz pulse propagation through the waveguide can be described as a linear superposition of the coupled propagating modes. The parameters of the waveguide, as well as the characteristics of the THz radiation, determine the conditions for modes coupling and thus the appearance of the output radiation, for instance, its chirp. More details about physical interpretation can be found in^29,34^. Figure 1(b,c) illustrates the experimental quasi-discrete spectrum and the temporal structure of the formed pulse train.

It was previously shown^26,27^ that a linearly chirped pulse interfering with itself shifted by a time delay shorter than its duration formed a pulse train which had a strict corresponding relation to its quasi-discrete spectrum. This means that each subpulse in the temporal structure has its own spectral line in the quasi-discrete spectrum. In this paper, due to the exponential chirp in the THz spectrum, the presence of the correspondence between the temporal and spectral structures is not obvious. In Fig. 1(b) three discernible pulse spikes can be seen. In the temporal structure Fig. 1(c) these spikes correspond to three pulses of different frequencies and duration. The formation of an irregular temporal sequence is associated with a nonlinear (exponential) pulse chirp. Figure 1(c) shows that the duration of individual peaks in the sequence varies from 10 to 40 ps, which corresponds to a repetition rate of 25–100 GHz. In this case, the chirp determines the correlation between the temporal and spectral structures^27^. Moreover, encoding can be carried out in the spectral domain, where we have a regular quasidiscrete structure Fig. 1(b) formed by two-beam interference^11^. Encoding in this case, should be done in the spectral domain by separating the spectrum in space and using amplitude filters, as it was previously shown in NIR range^26^.

## Discussion

To verify the assumptions made during the analysis of experimental data, a numerical simulation was carried out with parameters close to experimental ones. THz pulse with exponential chirp can be represented as:

1 $$E={E}_{0}\cdot \exp \left(-2\frac{{t}^{2}}{{\tau }_{0}^{2}}\right)\cdot \,\sin ({\omega }_{0}(1+\exp (-\alpha t))t)$$

where *E*_0_ is the pulse amplitude, *ω*_0_ = 2*πv*_0_ and *v*_0_ is the pulse central frequency, *τ*_0_ is the pulse duration, *α* is the inverse steepness of exponential chirp. To match the experiment, these parameters were chosen as follows: *v*_0_ = 0.45 THz, *τ*_0_ = 17 ps and *α* = 1/22 ps^−1^. The difference between pulse duration *τ*_0_ in numerical simulation and experiment is caused by the fact that a Gaussian pulse profile is used in the numerical simulation, while in the experiment the chirped THz pulse has an asymmetric non-Gaussian profile. Therefore, the pulse duration in the numerical simulation was increased to match the experimental pulse duration.

**Figure 2:**
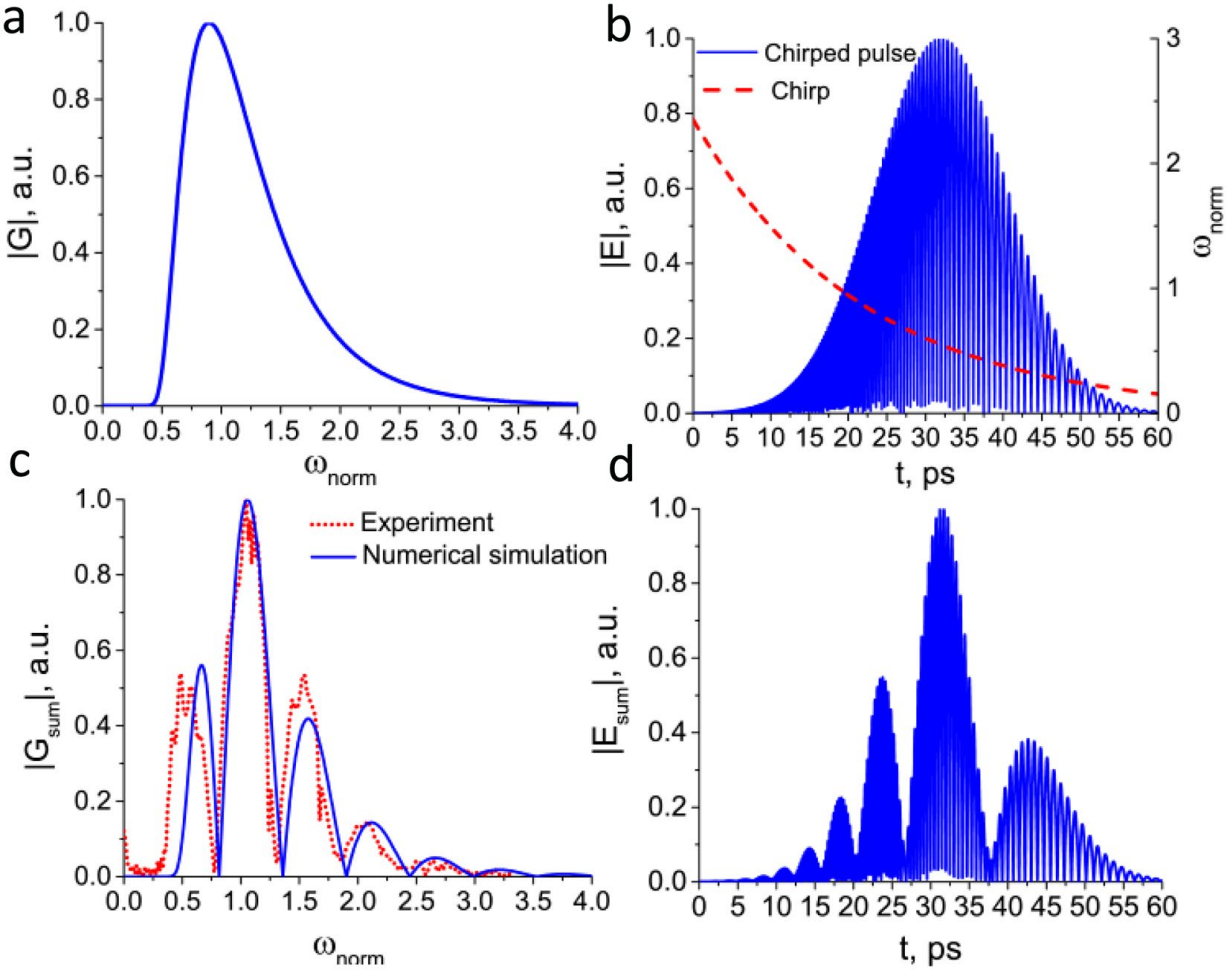
(**a**) Spectrum, (**b**) temporal structure and chirp of simulated chirped THz pulse. (**c**) Quasi-discrete spectrum and (**d**) pulse train formed from interference of two chirped pulses.

Figure 2(a,b) illustrates spectrum and temporal structure of the exponential chirped THz pulse obtained from numerical simulation. The interference of two pulses in temporal domain can be represented as^26,27^:

2 $${E}_{sum}=E(t)+E(t+\Delta t)$$

where Δ*t* is the time delay between pulses. Figure 2(c,d) represents such pulse interference with itself shifted by the time delay Δ*t* = 4 ps and the comparing of the corresponding quasi-discrete spectrum with experimental results (Fig. 2(c)). It can be seen that the simulated spectrum agrees well with the experimental one. As already noted, in the numerical simulation a Gaussian profile is used to describe the temporal structure. In the experiment, the temporal structure has an asymmetric non-Gaussian profile. In this regard, a comparison of these structures, as well as their interference is not indicative, since in the experiment asymmetric broadening with respect to the maximum occurs (see Fig. 5(c)), while the numerical simulation gives a symmetric broadening (see Fig. 2(b)).

**Figure 3:**
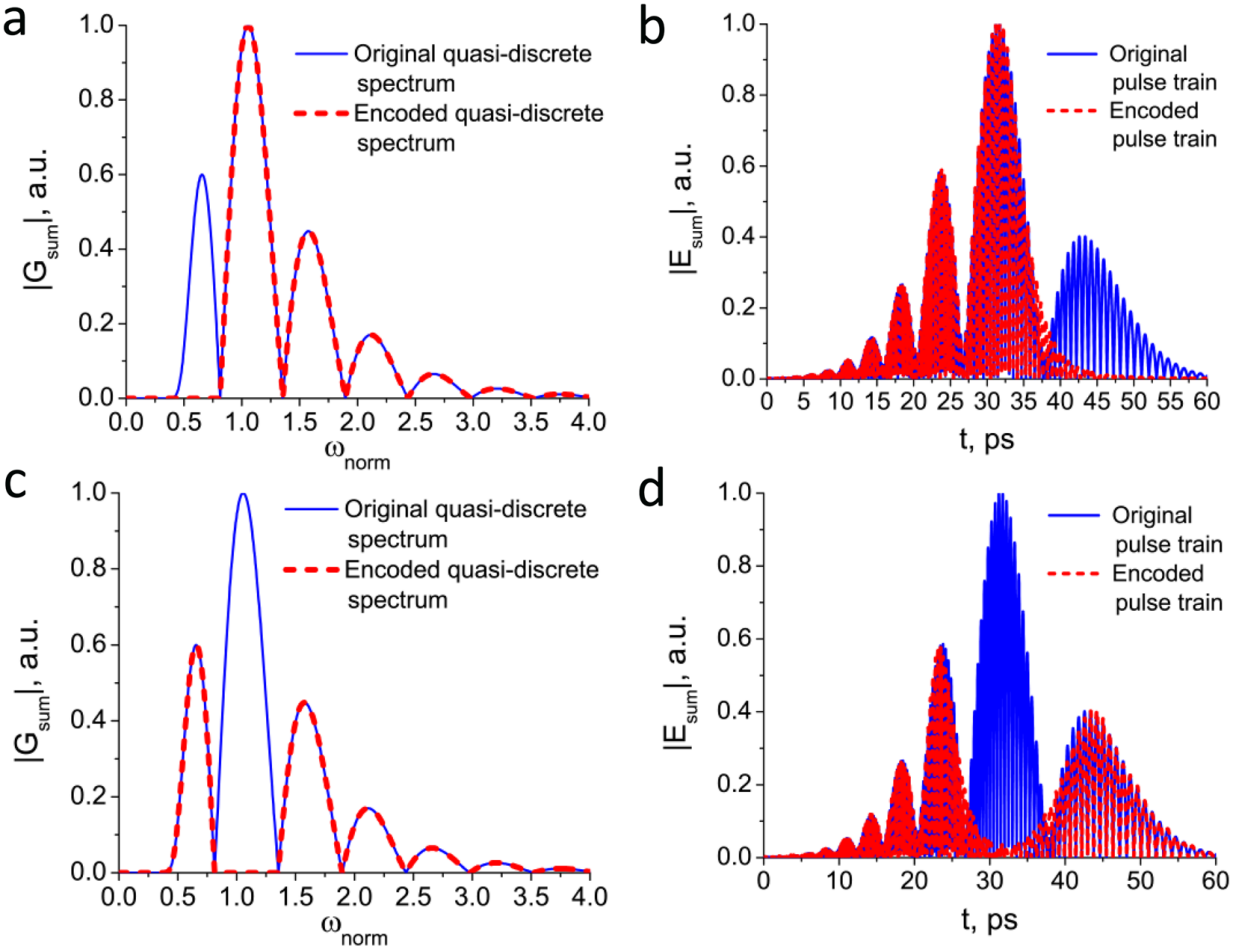
Comparison of original pulse (blue solid line) and encoded pulse (red dashed line) with cutting out one of the lines. (**a**), (**c**) Quasi-discrete spectrum and (**b**), (**d**) corresponding pulse train.

To confirm the correspondence between the temporal and spectral structures formed during the interference, a numerical simulation of the removal of one line in the quasi-discrete spectrum was performed. The results are shown in Fig. 3. It can be seen that cutting out one of the spectral peaks leads to the vanishing of the subpulse in temporal structure. However, there is some ambiguity in temporal domain which can be explained by the presence of a residual interference term. This term can be eliminated by the proper selection of experimental parameters^27^. Thus, changes in spectrum lead to similar changes in temporal domain. In other words there is a “linkage relation” between spectrum and temporal structure of THz pulse train. It should be noted that in the case of two-beam interference, the position of the minima of the quasidiscrete structure in the spectral region, which is determined by the time delay between pulses, is fixed. The absolute value of modulated spectrum is described by the following expression^11^:

3 $$|{G}_{sum}(\omega )|=|{G}_{0}(\omega \mathrm{)|(1}+cos(\omega \varDelta t))$$

where Δ*t* is a time delay between pulses and correspond to 3 ps in our experiment. The error associated with a double-pass of interferometer is equal to 2 *μ*m (~6.7 fs). Therefore the accuracy of the minima position in the spectrum can be calculated as the ratio of the aforementioned value to the delay between pulses to be 0.2%. In addition, as mentioned above encoding and decoding take place in the spectral range. The positions of spectral minima are fixed, and will not be affected by the instability of driving laser energy and its duration that leads to the changes in the temporal domain. As a result, this will not affect the transmitted information.

In order to further clarify the application prospects of this method, we provide the channel parameters (by channel we mean one of the peaks in the Fig. 2(c), e.g. the central one) of the original unoptimized communication system for the future establishment of the communication system. The ratio of the signal at the carrier frequency to noise (C/N) is of the order of 10^3^ or 30 dB. BER in the case of binary message sources, bit rate of 75 Mbit/s (which corresponds to the laser repetition rate of 75 MHz for binary message sources) and spectral channel width of 250 GHz is equal to 1.5 · 10^−7^. For 50 cm transmission, the link budget is calculated to be: Received Power (−94.7 dBm) = Transmitted Power (−15.2 dBm) + Gains (0 dB) - Losses (79.5 dB). Competitive with existing short-range THz CW channels (10^−3^–10^−7^ BER)^30–33^, the method is promising in further establishment of the communication system.

In conclusion, this paper has shown experimentally the feasibility of forming a variable sequence of THz subpulses with 25–100 GHz repetition rate in temporal domain. This is achieved by interference of two chirped THz pulses with a time delay less than their duration. As a result, the stable regular structure is formed in the spectral domain. The modulation frequency in the spectral and temporal structures is controlled by the time delay between the interfering pulses. Simulation-based results show that with the exponential chirped THz pulses, there is a “linkage relation” between the temporal and spectral structures. The data rate of such system is equal to the laser pulse repetition rate multiplied by the number of spectral peaks, in our case it is 75 · 3 = 225 Mbit/s. Estimated metrics of the communication channel based on the proposed method show that it is competitive with existing short-range THz CW channels. Due to active research both in the field of integrated chip lasers^35^, as well as in the development of miniature THz antennas^36,37^, this method of modulation, control and coding of broadband THz radiation in the temporal and spectral domain can be further improved and applied for short-range THz communication.

## Methods

**Figure 4:**
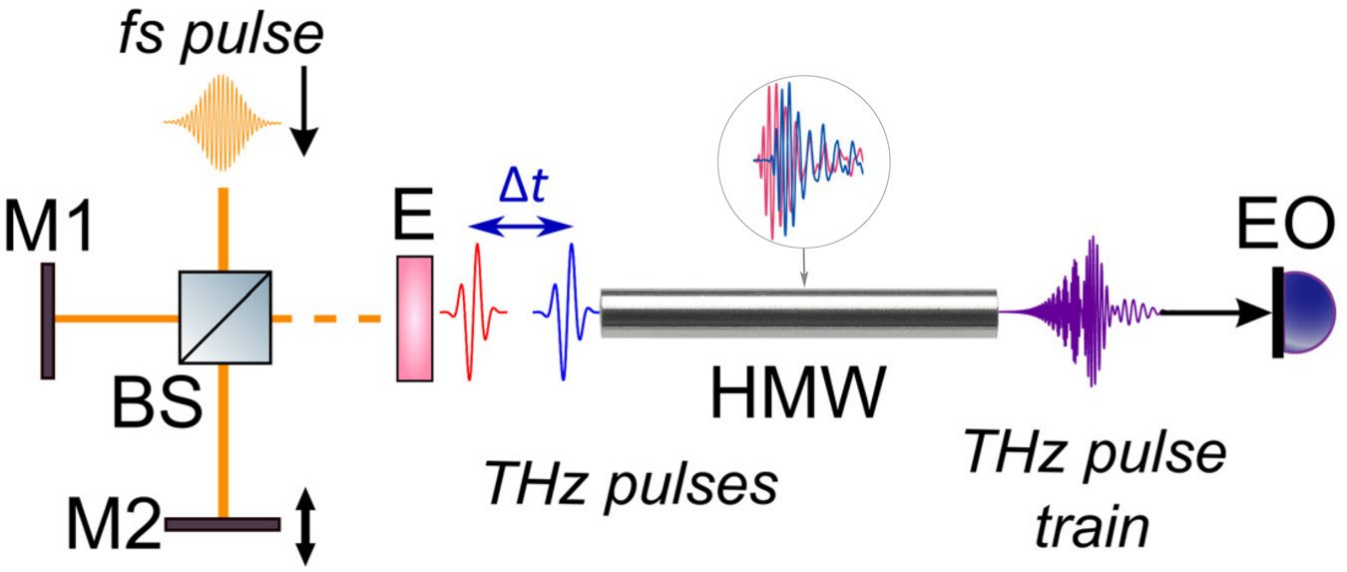
Experimental setup for THz pulse train generation from interference of two chirped THz pulses based on the conventional THz time-domain spectrometer. BS – beam splitter, M1, M2 – Michelson interferometer mirrors, E – THz emitter – InAs crystal for optical–to–THz conversion, HMW – hollow metal waveguide, EO – electro-optic detection system.

**Figure 5:**
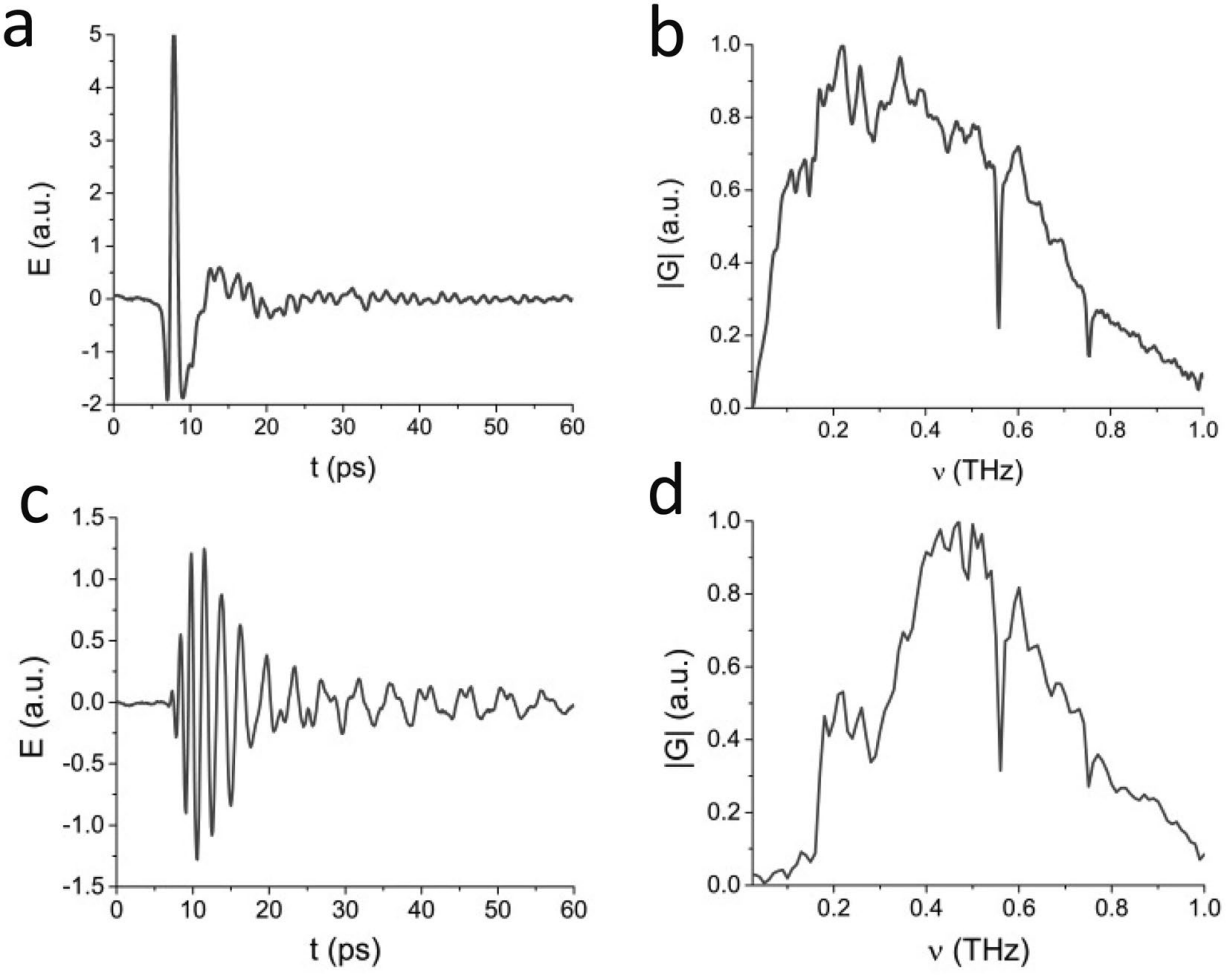
(**a**) Single THz pulse generated in InAs and (**b**) its spectrum. (**c**) The chirped THz pulse from hollow metal waveguide and (**d**) its spectrum.

Figure 4 illustrates the experimental setup for THz pulse train generation from interference of two chirped THz pulses based on the conventional THz time-domain spectrometer^38^. In this system, the THz radiation is generated due to the photo-Dember effect in an InAs crystal located in 2.4 T magnetic field^39^. The Yb-doped solid-state fs oscillator (central wavelength 1050 nm, duration 100 fs, pulse energy 70 nJ, repetition rate 75 MHz) is used as a pump. The THz radiation has central frequency 0.3 THz, estimated average power 30 *μ*W, and FWHM ~2 ps (see Fig. 5(a,b)). [100]-oriented CdTe crystal is used for electro-optical detection.

It is known that two-beam interference leads to quasi-discrete spectrum^11^. In our work Michelson interferometer in front of a THz generator is used to create two consecutive fs pulses. One of the mirror is fixed while the other is located on the linear stage which allow to adjust the time delay Δ*t* between fs pulses. The time delay is chosen so that the THz pulses generated in the InAs crystal do not interfere with each other. Then these pulses pass through the hollow metal waveguide^40^, where they overlap in time and interfere (see inset of Fig. 4). As the result of the interference THz pulsed train and corresponding quasi-discrete spectrum are generated (see Fig. 1(b,c)). The time delay Δ*t* with the fixed waveguide parameters allows to control the modulation in the temporal and spectral structures^25^.

Figure 5(a,b) show example of generated single THz pulse and its spectrum and Fig. 5(c,d) illustrate the chirped THz pulse obtained from hollow stainless steel metal waveguide with 23 mm length, 0.89 mm tip inner diameter, and 1.43 mm outer diameter.

As can be seen from the Fig. 5, chirping in a metal waveguide leads to an increase in the THz pulse duration from 2 ps to 7 ps (FWHM), while the corresponding spectrum shifts its central frequency to 0.45 THz and undergoes some minor changes. For example, at low frequencies there is a drop due to the fact that at these frequencies the signal does not propagate in the waveguide with such parameters. However, the water absorption line at a frequency of 0.55 THz remains unchanged. The 0.3 THz dip is also caused by the propagation features of THz radiation in the waveguide^29,40,41^.
